# A Feedforward Loop within the Thyroid-Brown Fat Axis Facilitates Thermoregulation

**DOI:** 10.1038/s41598-020-66697-0

**Published:** 2020-06-15

**Authors:** Lijuan Sun, Hui Jen Goh, Priya Govindharajulu, Lei Sun, Christiani Jeyakumar Henry, Melvin Khee-Shing Leow

**Affiliations:** 1 Singapore Institute for Clinical Sciences, Agency for Science, Technology and Research (A*STAR), Singapore, Singapore; 2 Singapore Institute of Food and Biotechnology Innovation, Agency for Science, Technology and Research (A*STAR), Singapore, Singapore; 3 Cardiovascular and Metabolic Disorders Program, Duke-NUS Medical School, Singapore, Singapore; 4 Department of Biochemistry, Yong Loo Lin School of Medicine, National University of Singapore (NUS), Singapore, Singapore; 5 Lee Kong Chian School of Medicine, Nanyang Technological University (NTU), Singapore, Singapore; 6 Department of Endocrinology, Tan Tock Seng Hospital (TTSH), Singapore, Singapore

**Keywords:** Physiology, Endocrinology

## Abstract

Thyroid hormones (TH) control brown adipose tissue (BAT) activation and differentiation, but their subsequent homeostatic response following BAT activation remains obscure. This study aimed to investigate the relationship between cold- and capsinoids-induced BAT activation and TH changes between baseline and 2 hours post-intervention. Nineteen healthy subjects underwent ^18^F-fluorodeoxyglucose positron-emission tomography (^18^F-FDG PET) and whole-body calorimetry (WBC) after 2 hours of cold exposure (~14.5 °C) or capsinoids ingestion (12 mg) in a crossover design. Standardized uptake values (SUV-mean) of the region of interest and energy expenditure (EE) were measured. Plasma free triiodothyronine (FT3), free thyroxine (FT4) and thyroid stimulating hormone (TSH) were measured before and 2 hours after each intervention. Subjects were divided into groups based on the presence (n = 12) or absence (n = 7) of BAT after cold exposure. 12 of 19 subjects were classified as BAT-positive. Subjects with BAT had higher baseline FT3 concentration, baseline FT3/FT4 ratio compared with subjects without BAT. Controlling for body fat percentage, FT3 concentration at baseline was associated with EE change from baseline after cold exposure (P = 0.037) and capsinoids (P = 0.047). Plasma FT4 level significantly increased associated with reciprocal decline in TSH after acute cold exposure and capsinoids independently of subject and treatment status. Circulating FT3 was higher in BAT-positive subjects and was a stronger predictor of EE changes after cold exposure and capsinoids in healthy humans. BAT activation elevates plasma FT4 acutely and may contribute towards augmentation of thermogenesis via a positive feedback response.

## Introduction

Thyroid hormones (TH) including triiodothyronine (T3) and thyroxine (T4) have long been well established to play an important role in energy expenditure (EE) and basal metabolic rate^1^. Regulating thermogenesis is one of the major tasks of TH in adult humans^2^. TH are important for heat generation during shivering and non-shivering cold adaptation. Furthermore, oxygen consumption and heat production at rest and during activities are sensitive to small alterations of TH^3^. The mechanisms for thyroid thermogenesis are complex^4^ and not fully elucidated, but one of its key thermogenic effectors operate through brown adipose tissue (BAT)^5^. With respect to thermoregulatory control, BAT is a critical organ that evolved primarily in mammals to defend against hypothermia through non-shivering thermogenesis (NST). The physiological link between BAT and TH is regulated by the sympathetic nervous system (SNS), while the molecular basis linking TH and SNS to BAT is now understood to operate via the 5’-flanking region of the uncoupling protein 1 (UCP1) gene under the control of both thyroid hormone response element (TRE) and cAMP response element (CRE)^4,6,7^. Effective adaptive thermogenesis in BAT requires TH^8^, adrenergic stimulation, and uncoupling protein-1 (UCP-1) expression^9^. TH mediation of adaptive thermogenesis involves both thyroid hormone receptor-α (TRα) and –β (TRβ). While the sympathetic response of BAT depends on TRα, Ribeiro *et al*.^9^ reported that TRβ is required for UCP-1 dependent adaptive thermogenesis in BAT of mice that underscored the importance of TH for BAT function. Lopez *et al*.^10^ reported that TH are able to up-regulate BAT via the brain as central administration of triiodothyronine (T3) resulted in BAT activation in rats. Adequate circulating concentrations of unbound, free T3 (FT3) derived from intrathyroidal and peripheral deiodination of T4 are necessary for recruitment of brown adipocytes and up-regulation of UCP-1. More recently, we have demonstrated that T3 also contributes to BAT activation via mitochondrial biogenesis and MTOR-mediated mitophagy^11^. T3 is thus one of the key essential hormones in the process of BAT activation and browning^12–14^.

Cold stress increases the expression and activity of tissue cAMP-dependent type 2 iodothyronine deiodinase (DIO2), a selenoenzyme which stimulates the conversion of T4 to active T3^15^. TH has been extensively shown to play an important role in cold adaptation in rodents and humans^16^. As TH can influence BAT mass and activity with resulting augmentation of white adipose tissue (WAT) fat catabolism^17,18^, then TH probably exert a physiological role in mitigating against obesity and metabolic syndrome. Conversely, while NST to preserve core temperature is a primary function of BAT activation, it remains unknown if active BAT in turn affects thyroidal TH secretion through some form of homeostatic feedback. In our previous report, we found that EE was significantly increased after cold exposure and capsinoid ingestion in BAT-positive subjects assessed through ^18^F-FDG PET^19^. Although this can be attributed purely to BAT activation, might the thyroid also contributed to the persistence and maintenance of EE elevation via TH? To gain further insights regarding BAT activation effects on TH and vice versa in adult humans, this study evaluated TH changes after cold and capsinoids exposure in BAT-positive and BAT-negative healthy participants. The participants’ BAT status was classified by BAT activity via ^18^F-FDG PET/MR scan. The relationship between BAT and TH before and after cold and capsinoids exposure were evaluated.

## Subjects and Methods

### Subjects

Twenty-two healthy adults were recruited in this study. 2 subjects dropped out during the study due to unanticipated claustrophobia during PET/MR scan and one subject failed venous cannulation attempts before and after the whole body calorimetry (WBC) session and were thus excluded; 19 of them (age: 21–35 years; BMI: 18.5–25.9 kg/m^2^) completed all study procedures (Table 1). All participants were healthy and none had any personal or family history of either type 2 diabetes mellitus, thyroid or cardiovascular disease. None of the subjects smoked or used tobacco products, consumed special diets, or took medications known to alter BAT metabolism. All subjects were euthyroid with normal serum free thyroxine (FT4), FT3 and TSH concentrations. The present study was conducted according to the ethical guidelines of the Declaration of Helsinki, and all procedures were approved by the Domain-Specific Review Board of National Healthcare Group, Singapore (DSRB approval reference: 2015/00715), and registered with ClinicalTrials.gov (NCT02964442). Written informed consent was obtained from all subjects before participation.

**Table 1:** Characteristics of total participants, participants with and without BAT after cold exposure.

	Total (19)	BAT-positive (12)	BAT-negative (7)	P value
Age (years)	26.0 ± 1.0	26.9 ± 1.2	24.4 ± 1.4	0.22
BMI (kg/m^2^)	21.7 ± 0.6	21.7 ± 0.8	21.8 ± 0.9	0.95
Body fat (%)	29.7 ± 1.8	29.4 ± 2.8	30.1 ± 1.5	0.85
Fat mass (kg)	18.6 ± 1.8	19.2 ± 2.7	17.6 ± 1.0	0.67
Lean body mass (kg)	40.9 ± 2.1	42.0 ± 2.7	39.2 ± 3.5	0.53
RMR (kcal/day)	1569 ± 75	1574 ± 88	1561 ± 145	0.94
Triglyceride (mmol/L)	0.9 ± 0.06	1.0 ± 0.1	0.7 ± 0.1	0.04
TSH (mIU/L)	2.1 ± 0.2	2.3 ± 0.3	1.7 ± 0.2	0.24
FT4 (pmol/L)	16.9 ± 0.6	17.1 ± 0.8	16.6 ± 0.7	0.68
FT3 (pmol/L)	4.8 ± 0.1	5.0 ± 0.1	4.3 ± 0.2	0.008
Free T3/Free T4 ratio	0.3 ± 0.01	0.3 ± 0.01	0.26 ± 0.01	0.047
EE 2 h (kcal/day)	1778 ± 82	1841 ± 101	1673 ± 139	0.34
∆ EE (kcal/day)	210 ± 27	267 ± 29	111 ± 24	0.002
PET SUV	2.1 ± 0.3	2.9 ± 0.2	0.8 ± 0.1	<0.001

Data presented as mean ± SEM. P values represents Student’s t-test between BAT- positive and BAT- negative participants. BAT, brown adipose tissue; BMI, body mass index, was calculated as body weight (kg) divided by the square of height (m); RMR, resting metabolic rate; TSH, thyroid-stimulating hormone; FT4, free thyroxine; FT3, free triiodothyronine; EE, energy expenditure; PET, positron emission tomography; SUV, standardized uptake value.

### Study design

The subjects attended two separate visits for PET/MR and two separate visits for whole body calorimetry /infrared thermal imaging (WBC/IRT), to assess the effects of cold exposure and capsinoids ingestion. Visits were separated by a minimum of 48 hours washout period in order to minimize carryover effects. Capsinoids capsules (12 mg, 8 gel capsules) were provided by Ajinomoto Inc. (Tokyo, Japan). The composition details were described previously^20^. Cold exposure was achieved using a cooling vest with a fixed temperature of 14.5 °C (Cool 58, Polar Products, Ohio, US). The participants were asked to refrain from caffeine, alcohol intake and any vigorous physical activity on the day before the study. The study protocol and flow chart were described previously^19^.

### ^18^F-FDG PET/MR Imaging

After fasting for 10–12 hours, the participants arrived at the Clinical Imaging Research Centre. An indwelling cannula was inserted into a forearm vein by a registered nurse after a 10-min rest, and fasting blood glucose was measured. At the first visit, subjects were instructed to ingest capsinoids 30 minutes before the PET/MR scan. During the second visit, subjects donned on the cold vest upon arrival at the research centre. For both visits, subjects were administered an intravenous injection of ^18^F-FDG (3 mCi), 20 min before the start of the scans. The scans were performed on a hybrid PET-MR system (Biograph mMR, Siemens Healthcare, Erlangen, Germany) for 80 minutes. One experienced researcher blinded to the allocated intervention assessed FDG uptake on both sides of neck and supraclavicular regions excluding bone, muscle and blood vessels. BAT activity was quantified by calculating SUV mean, SUV max and BAT volume. The cut-off value of SUV mean for dividing subjects into BAT-positive and BAT-negative groups was 2.0. Detectable BAT volume was calculated as the sum of pixels meeting this classification criteria multiplied by the pixel volume. The imaging analysis was described as previously^21^.

### Whole body calorimetry

After an overnight fast of 10–12 hours, EE was assessed through gaseous exchanges using a dual-chamber whole body calorimeter (WBC) facility located at the Clinical Nutrition Research Centre. The WBC chambers are open-circuit air-tight indirect calorimeters and provide EE, fat oxidation and respiratory quotient (RQ) measurement based on oxygen consumption and carbon dioxide production. The WBC has been described in detail in our previous published paper^22^. During each test session, after 45 minutes of resting metabolic rate (RMR) measurement, the subjects were asked to wear the cooling vest or ingest 12 mg capsinoids inside the WBC room for up to 2 hours.

### Blood analysis

Screening blood samples were sent to the National University Hospital referral laboratory for biochemical assessment of renal, liver and thyroid function. Fasting and 2 hours post-intervention blood samples during the WBC session was collected from venous samples to measure TH. Plasma concentration of free TH (FT3, FT4) and TSH were measured using immunoassay analyser (Cobas e411, Roche). The intra**-**assay coefficient of variation (intra**-**assay CV) of the Cobas immunoassays for the analytes was less than 3%.

### Data and statistical analysis

This was a secondary analysis of data acquired from participants in a protocol that investigated BAT activation and energy expenditure by cold and capsinoids stimulation (www.Clinical Trials.gov identifier NCT 02964442). Details on power calculations were shown in our previous publication^23^. EE was calculated based on volume of O_2_ consumption (VO_2_) and CO_2_ production (VCO_2_) using the Weir equation^24^. RMR was defined as the average 45 minutes EE during the 45 min measurement period. The change in EE and fat oxidation (FOX) in 2 hours was calculated as average 2 hours EE and FOX minus RMR. Paired-t tests were used to compare the differences between baseline and 2 hours post-intervention in the concentrations of free, unbound circulating TH. The data were checked for normality using Shapiro-Wilk test and visually using histogram and Q-Q-plot. The data analyzed by parametric statistics were normally distributed. Pearson correlations were performed to identify relations between variables. Data were presented as means ± SEM, unless otherwise stated. A P-value ≤ 0.05 was considered statistically significant. Statistical analysis was performed by using SPSS software version 23 (IBM SPSS Inc.).

## Results

### Clinical characteristics

The baseline characteristics of the 19 subjects are summarized in Table 1. Cold exposure increased the activity and volume of detectable BAT in 12 subjects. BAT was found in the cervical and supraclavicular adipose depots, with an estimated average SUV mean of 2.9, SUV max of 7.9 and average BAT volume of 75 cm^3^ after cold exposure (Table 1). Following the ^18^F-FDG uptake post-cold exposure measured by calculating mean SUV, the subjects were divided into two groups: those with no detectable ^18^F-FDG uptake (BAT-negative, n = 7) and those with detectable ^18^F-FDG uptake with mean SUV ≥ 2 (BAT-positive; n = 12). No significant differences in anthropometric measurements were found between the two groups (Table 1). However, we found fasting baseline FT3 level significantly higher in BAT-positive subjects (5.0 ± 0.1) compared with BAT-negative subjects (4.3 ± 0.2) (P = 0.008). The ratio of fasting baseline FT3/FT4 was significantly higher in BAT-positive subjects as compared with BAT-negative subjects (P = 0.047). No significant difference was found for TSH (P = 0.24) and FT4 (P = 0.68) levels between BAT-positive subjects and BAT-negative subjects in fasting basal state.

### Relationship of thyroid hormones, blood lipids and body composition

Fasting FT3 concentration was positively associated with lean body mass (r = 0.49, P = 0.04), REE (r = 0.55, P = 0.01) and triglyceride level (r = 0.56, P = 0.01) but not with other baseline measurements (Table 2). FT4 concentration was negatively associated with % fat mass (r = −0.56, P = 0.01) and positively associated with fasting glucose level (r = 0.46, P = 0.047) but not with other baseline metabolic measurements including lean body mass (P = 0.20) and triglycerides (P = 0.59). TSH was not associated with baseline characteristics we have measured in Table 2.

**Table 2:** Correlations between fasting thyroid hormone levels, body composition, blood markers and resting energy expenditure in total participants.

Measure	FT3 (pmol/L)		FT4 (pmol/L)		TSH (uIU/mL)	
	Coefficient (r)	p-value	Coefficient (r)	p-value	Coefficient (r)	p-value
BMI (kg/m^2^)	0.32	0.18	−0.04	0.87	−0.21	0.4
Lean body mass (kg)	**0.49**	**0.04**	0.31	0.20	0.06	0.82
% fat mass	−0.21	0.38	**−0.56**	**0.01**	−0.29	0.24
Visceral fat	0.47	0.07	−0.05	0.85	−0.14	0.60
REE (kcal/day)	**0.55**	**0.01**	0.26	0.28	−0.02	0.92
Glucose (mmol/L)	0.32	0.19	**0.46**	**0.047**	−0.24	0.33
Triglyceride (mmol/L)	**0.56**	**0.01**	−0.13	0.59	0.15	0.55

N = 19; TSH, thyroid stimulating hormone; FT4, free thyroxine; FT3: free triiodothyronine; BMI, body mass index; REE, resting energy expenditure.

### Cold and capsinoids-induced thyroid hormones changes

In all of the participants, both cold exposure and capsinoids ingestion significantly increased FT4 concentrations coupled with decreased TSH concentrations compared with baseline (Table 3). There were similarities between the effects of cold exposure and capsinoids ingestion on FT4 and TSH. The increase of FT3 was significantly higher after cold exposure compared with capsinoids ingestion (Table 3). However, when compared with baseline, 2 hours of cold exposure did not result in any significant change in FT3 level in all subjects which means BAT only affects baseline FT3 concentration with only small changes following BAT activation by cold within the same subjects (Table 3). By contrast, FT4 concentration was significantly increased after 2 hours of cold or capsinoids exposure regardless of BAT status (Table 4). TSH concentration was significantly decreased after 2 hours of cold exposure in all participants independent of BAT status (Table 4).

**Table 3:** Thyroid hormones concentrations at baseline and 2 h post intervention and change from baseline after 2 treatments^1^.

	Capsinoids			Cold exposure		
	baseline	120 min	∆ change	baseline	120 min	∆ change
TSH (uIU/mL)	2.2 ± 0.3	1.8 ± 0.2^2^	−0.4 ± 0.1	2.1 ± 0.2	1.6 ± 0.2^2^	−0.5 ± 0.1
FT4 (pmol/L)	16.4 ± 0.6	16.9 ± 0.6^2^	0.5 ± 0.1	16.9 ± 0.6	17.2 ± 0.6^2^	0.3 ± 0.1
FT3 (pmol/L)	4.7 ± 0.2	4.7 ± 0.2	−0.04 ± 0.04	4.8 ± 0.2	4.8 ± 0.2	0.05 ± 0.04^3^

^1^Values are means ± SEMs; n = 19. TSH, thyroid stimulating hormone; FT4, free thyroxine; FT3: free triiodothyronine.

^2^Different from baseline within the same treatment group, P < 0.05. ^3^Different from after capsinoids ingestion, P < 0.05.

**Table 4:** Summary of outcomes of thyroid hormones after capsinoids ingestion and cold exposure in BAT positive and BAT-negative subjects^1^.

	BAT-positive subjects				BAT-negative subjects			
	Capsinoids ingestion		Cold exposure		Capsinoids ingestion		Cold exposure	
	baseline	120 min	baseline	120 min	baseline	120 min	baseline	120 min
TSH (uIU/mL)	2.4 ± 0.4	2.0 ± 0.3^2^	2.3 ± 0.3	1.8 ± 0.3^2^	1.9 ± 0.2	1.5 ± 0.3^2^	1.7 ± 0.2	1.3 ± 0.1^2^
FT4 (pmol/L)	16.7 ± 0.9	17.2 ± 0.9^2^	17.1 ± 0.8	17.5 ± 0.8^2^	15.9 ± 0.7	16.4 ± 0.7^2^	16.6 ± 0.7	16.8 ± 0.7^2^
FT3 (pmol/L)	5.1 ± 0.2	5.0 ± 0.2	5.0 ± 0.1	5.1 ± 0.1	4.1 ± 0.3	4.1 ± 0.3	4.3 ± 0.2	4.4 ± 0.3

^1^Values are mean ± SEMs; all values calculated from 19 subjects (12 BAT-positive subjects vs. 7 BAT-negative subjects); ^2^Different from baseline within the same treatment group, P < 0.05; TSH, thyroid stimulating hormone; FT4, free thyroxine; FT3: free triiodothyronine.

### Relationship of BAT, EE and thyroid hormones

**Figure 1:**
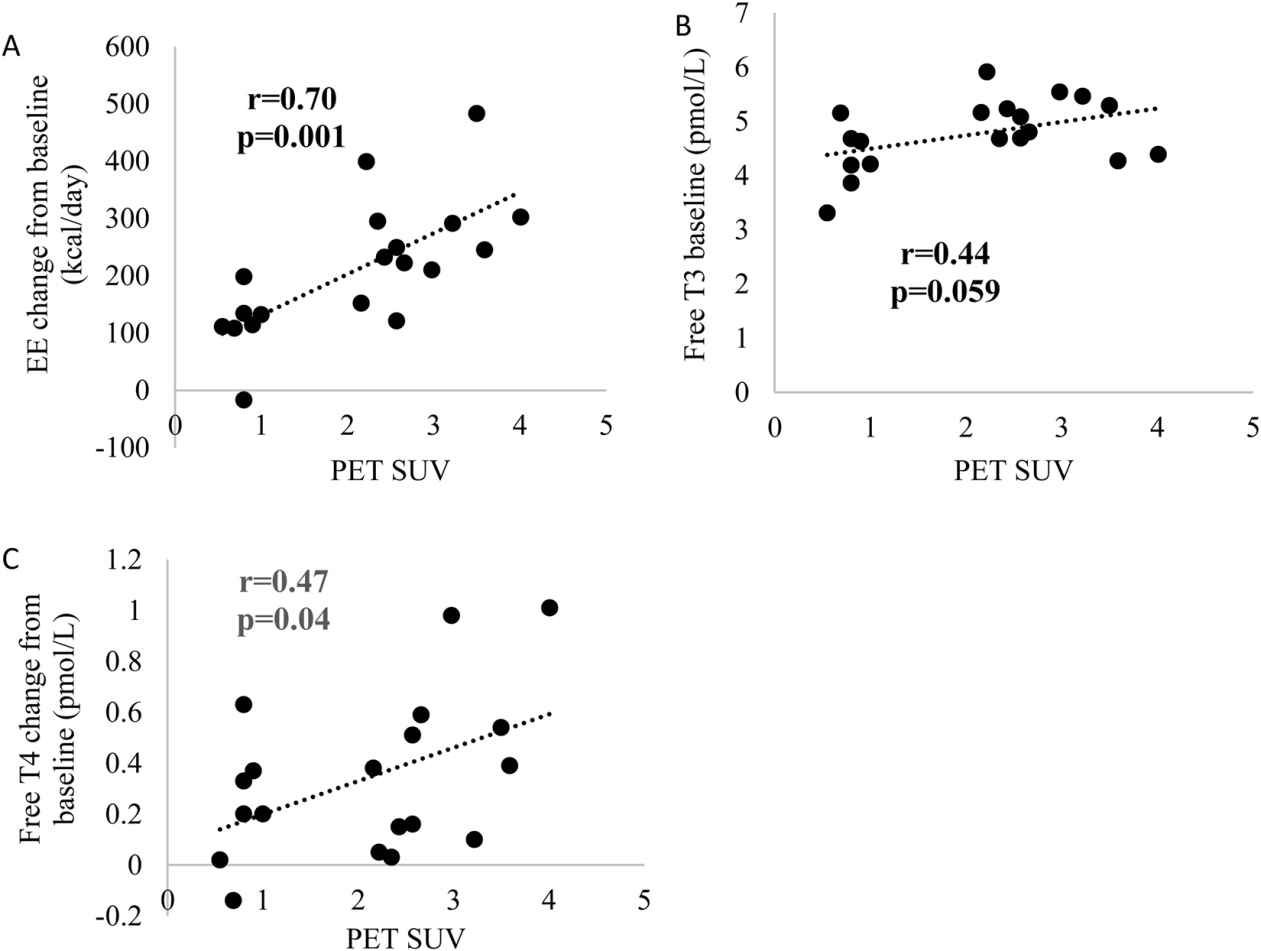
Correlations between PET SUV and EE change from baseline after cold exposure (**A**), FT3 at baseline (**B**) and FT4 concentration change from baseline after cold exposure (**C**) (n = 19). PET, positron emission tomography; FT3, free triiodothyronine; FT4, free thyroxine.

**Figure 2:**
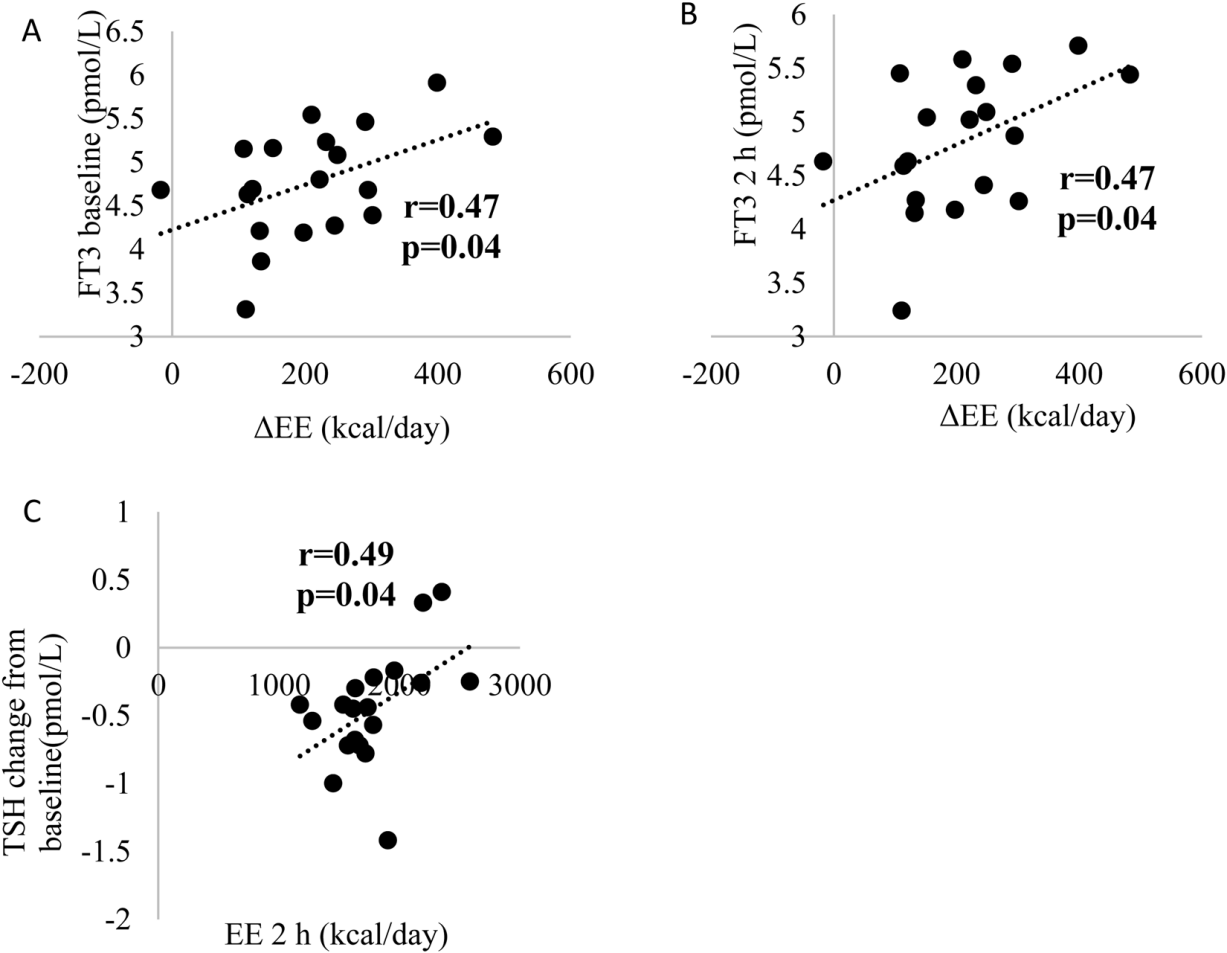
Correlations between EE change from baseline (ΔEE) and FT3 at baseline (**A**) and 2 hours post-cold exposure (**B**), TSH change from baseline and EE 2 hours after cold exposure (**C**) (n = 19). EE, energy expenditure; FT3, free Triiodothyronine; TSH, thyroid stimulating hormone.

Cold-induced BAT activity was measured via PET/MR as previous reported^25^. PET SUV was positively associated with EE change from baseline after cold exposure (r = 0.7, P = 0.001). There was a trend towards a positive association between FT3 concentration at baseline and PET SUV (r = 0.44, P = 0.06). Also, FT3 at baseline is correlated to EE change post-cold and post-capsinoids (Table 5). FT4 change from baseline after cold exposure was positively associated with PET SUV (r = 0.47, 0.04) (Fig. 1). But FT4 at baseline was not associated with EE change post-cold and post-capsinoids (data not shown). FT3 concentration at fasting and 2 hours after cold exposure were positively associated with EE change from baseline (both r = 0.47, P = 0.04). TSH change from baseline was positively associated with EE after 2 hours cold exposure (Fig. 2).

**Table 5:** Regression results of EE change from baseline with thyroid hormones at baseline and 2 hours after capsinoids ingestion and cold exposure.

baseline	∆EE after capsinoids ingestion			Coefficient	∆ EE after cold exposure	
	Coefficient	95% CI	P value		95% CI	P value
FT3 (pmol/L)	44.1	0.7, 87.5	**0.04**	73.4	−8.7, 155.5	**0.04**
FT4 (pmol/L)	1.5	−14.1, 17.1	0.8	−4.2	−31.1, 22.8	0.75
TSH (uIU/mL)	12.5	−17.7, 42.6	0.4	−6.2	−65.0, 52.5	0.83
120 min FT3 (pmol/L)	−4.6	−167.6, 158.4	0.95	−32.1	−387.9, 323.7	0.85
FT4 (pmol/L)	−38.7	−124.1, 46.9	0.35	60.0	−126.4, 246.3	0.50
TSH (uIU/mL)	10.2	−66.5, 86.8	0.78	94.2	−106.5, 294.9	0.33

All values calculated from 19 subjects. The baseline analysis was adjusted with body fat percentage (%). The 120 min analysis was adjusted with body fat percentage (%) and thyroid hormones baseline value. TSH, TSH, thyroid stimulating hormone; FT4, free thyroxine; FT3: free triiodothyronine.

## Discussion

The present findings revealed very interesting biological phenomena that have never previously been reported anywhere thus far in the extant literature. Essentially, we can better comprehend these TH changes when we scrutinize the results above at four levels in terms of group comparisons based on: (1) BAT status, (2) modality of BAT stimulation (ie. cold versus capsinoids), (3) pre-stimulated versus post-stimulated time points, and (4) FT3 versus FT4.

Firstly, two potential physiological processes may account for the observation of baseline TH levels (especially FT3) being significantly higher among BAT-positive compared to BAT-negative subjects. One hypothesis is that the presence of BAT within an individual accentuates TH secretion by the thyroid gland via some yet undefined positive feedback interactions and mechanisms. The other possibility is that a higher TH output by the thyroid results in greater brown adipocyte differentiation and BAT activation, which in turn increases the probability of higher TH being found among those who are BAT-positive. Both processes are not mutually exclusive and may co-exist in any given individual to produce this finding. It would require dynamic experiments using appropriately labelled tracers to decipher the kinetics involved and which mechanism predominates. Secondly, while both cold and capsinoids stimulation evoke changes in circulating TH levels regardless of BAT status, the magnitude of increase in TH was greater in FT4 compared to FT3. Because T4 is specifically produced by the thyroid whereas T3 can be derived from both the thyroid and peripheral tissues possessing 5’-deiodinase activity, it is likely that cold and capsinoids directly act on the SNS which then triggers the secretion of thyroidal secretion of TH, with T4 predominating over T3 as per established thyroid physiology^26^. As well, the TH elevations observed were consistently associated with significant reciprocal TSH reductions, which implies that the primary ‘driver’ of the TH secretion resides within the thyroid gland itself rather than being mediated at the central, hypothalamus-pituitary level.

Given that TSH is an exquisitely sensitive biomarker and regulator of ambient TH levels, the fact that TSH declined inversely to the TH escalations was itself remarkable as it suggested that these “quantitatively miniscule” changes in TH are physiologically significant. This crucial result implied, as a necessary corollary, that TSH should not have altered to any detectable extent had the magnitude of those TH changes been clinically irrelevant. Notably, the pattern of TSH change was similar in both cold and capsinoids stimulation of BAT, and TSH varied inversely with all these TH elevations.

Apparently, FT4 rose much more so than FT3 as a result of cold stimulation, associated with a decreased TSH. This is consistent with the fact that at any time, the secretory rate of T4 by the thyroid is nearly 20-fold higher than that of T3^27^. Apart from differences in thyroidal biosynthetic rate of T4 and T3, it is also known that the thyroid gland harbours a reservoir pool of preformed TH consisting of far more T4 and T3^28^. Hence, it is conceivable that the FT4 level would escalate far more than FT3 in response to any stimulus that increases net thyroid glandular secretory output of TH. Importantly, we observe that the increase in FT3 in the circulation evoked by cold and capsinoids regardless of BAT status is miniscule compared to FT4 despite the documented 10- to 50-fold upregulation of type 2 iodothyronine deiodinase (D2) selenoenzyme during cold stress in BAT^15^. This is probably because the accentuation of D2 activity led to locally generated T3 that is mainly intracellular within BAT adipocytes with much lesser T3 being secreted into the blood. On the other hand, as the activity of T3 is 3–8-folds higher than T4^29^, this could explain the strong correlation of FT3 changes to EE whereas FT4 changes showed no obvious correlation to EE.

**Figure 3:**
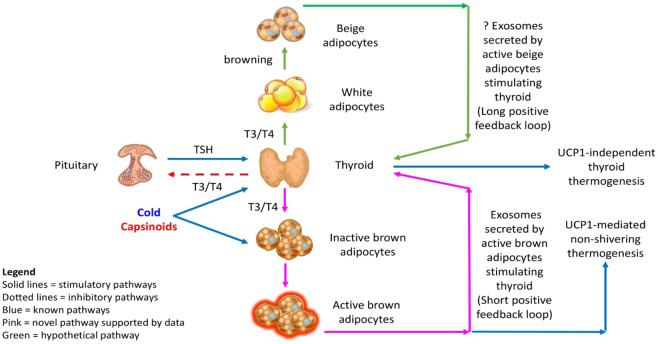
The positive feedback loops that amplify thermogenesis within the framework of a novel BAT-thyroid axis. BAT, brown adipose tissue; T3, triiodothyronine; T4, thyroxine; TSH, thyroid stimulating hormone; UCP1, uncoupling protein 1.

Interestingly, the fact that cold and capsinoids were also capable of inducing changes in TH and TSH regardless of BAT status supports yet another important notion, namely that such external stimuli might also directly act on the thyroid gland to release TH independently of BAT as well. As sympathetic innervation occurs within the thyroid follicles and norepinephrine released by intrathyroidal adrenergic nerves stimulates secretion of TH, it is not surprising that both cold and capsinoids which act via the SNS can stimulate an increase in TH with reciprocal reduction in TSH secretion even among those without demonstrable BAT^30^. Nevertheless, the finding that the increase in FT4 is 2.3-folds greater in BAT-positive relative to BAT-negative subjects seemingly support the presence of a tentative undefined feedforward factor emerging from activated BAT which has a capacity for stimulating T4 secretion. Finally, as the changes in TH and TSH were all significantly correlated to both PET-SUV and EE, our discovery lends credence to the hypothesis that the positive feedback loop bridging between the thyroid gland and BAT is not trivial but reveals an intriguing aspect of homeostatic regulation that probably plays a definite role in normal thermoregulation biology and in the maintenance of metabolic health. This shows that both BAT and the thyroid, being organs hardwired for thermogenesis, can be simultaneously activated independently by appropriate external stimuli with ensuing TH/TSH changes that reflected the existence of an intrinsically positive homeostatic feedback loop between the two organs (Fig. 3).

The mechanisms behind how BAT communicate with the thyroid likely consist of signalling pathways orchestrated via “batokines” (ie. BAT-derived adipokines) delivered to the thyroid packaged in exosomes. The recent literature has also shown that BAT exosomes contain non-coding RNA such as microRNAs and long non-coding RNAs which can exert gene regulation effects on distant targets, including the thyroid^31–35^. BAT-derived exosomes are also cellular cargoes for a wide range of proteins characterizing the BAT secretome. This BAT proteome can act as hormones and serve as biochemical mediators forming one limb of the feedforward path of the BAT-thyroid axis to generate an increased thyroidal output of TH. In this connection, it has been shown that neuregulin-4 (NRG4) is a novel adipokine secreted by both classical brown and beige adipocytes^36,37^. Plasma NRG4 levels have also been shown to be inversely related to the risk of obesity, metabolic syndrome and type 2 diabetes^36,38–40^. Interestingly, serum NRG4 has been demonstrated recently to be elevated in hyperthyroidism, a clinical state of heightened fat catabolism^41^. Hence, BAT exosomal NRG4 may potentially be one of several critical links between BAT and the thyroid gland^42^. Our own research also revealed the presence of selenoprotein P within cold-stimulated BAT exosomes (unpublished data). This finding adds further evidence that suggests BAT may regulate thyroid hormone synthesis because selenoprotein biosynthesis defects were shown to cause abnormal thyroid hormone production^43–45^. Such exosomal batokines may augment TH output via the observed short (acute) BAT-thyroid feedback loop mediated by pre-existing brown fat, and possibly a long (chronic) positive feedback loop mediated by beige fat derived from browning of WAT (Fig. 3). The consequent amplification in TH in this schema constitutes the other positive feedback path of the BAT-thyroid axis to reinforce BAT mass and activation. Conversely, while still hypothetical, the thyroid may equally serve as a source of exosomes that carry molecular cargoes critical for BAT activation and browning of WAT so as to accentuate thermogenesis. We have demonstrated previously that lnc-dPrdm16 is one such long non-coding RNA that mediates brown adipocyte differentiation^46^. It is thus conceivable that exosomal lnc-dPrdm16 from metabolically active tissues such as the thyroid could also regulate WAT browning and contribute to thermogenesis.

It may be argued that this novel axis may seem functionally negligible in humans living in persistently warm climates. However, we have elegantly shown here^19,25,47^ that a substantial number of people living within the tropics/equatorial belt with ambient temperatures up to 35 degrees Celsius can still be demonstrated to possess much activatable BAT by cold or capsinoids. Hence, we surmise that this BAT-thyroid axis is physiologically critical in maintaining normal metabolism in addition to defending against hypothermia. While speculative at this point, it is possible that a defect in this BAT-thyroid axis could be medically impactful and predispose the affected individuals to metabolic disorders, such as obesity and type 2 diabetes mellitus. No additional PET-MR scan at ambient temperature was performed on any subjects as a control condition due to ethical constraints and the need to abide by the ALARA principle of keeping ionization radiation exposure to the minimum while fulfilling any clinical/research objective for which we hereby acknowledge as a limitation of our study.

In summary, we postulate that part of the purpose for activated BAT triggering an increase in TH output by the thyroid gland is an evolutionary adaptation as such a biological response should translate, in accordance to fundamental control systems engineering principles, to a far more efficient and optimized thermogenic system if the resulting higher TH output in turn augments BAT activity and development (classic brown fat and beige fat via browning of WAT). We therefore predict that a similar ‘reflex’ feedback loop ought to occur in other mammalian vertebrates of comparable biology, a hypothesis awaiting further proof by future research. This positive feedback cycle within a BAT-thyroid axis favours the successful sustenance of thermogenesis and metabolic homeostasis more robustly than either organ operating independently without any linkage. Therefore, such a BAT-thyroid interaction conceivably exists to confer a biological survival advantage.

### Clinical trial registration

The trial was registered at clinicaltrials.gov as NCT02964442.
